# Timing and outcome prediction of intravenous thrombolysis in posterior circulation stroke: Insights from the Austrian Stroke Unit Registry

**DOI:** 10.1093/esj/23969873251341770

**Published:** 2026-01-01

**Authors:** Alexander Tinchon, Dominika Mikšová, Wilfried Lang, Stefan Krebs, Elisabeth Freydl, Christian Baumgartner, Oliver Friedrich, Stefan Oberndorfer, Marek Sykora

**Affiliations:** Karl Landsteiner University of Health Sciences, Krems, Austria; Division of Neurology, University Hospital St. Pölten, St. Pölten, Austria; Karl Landsteiner Institute of Clinical Neurology and Neuropsychology, University Hospital St. Pölten, St. Pölten, Austria; Gesundheit Österreich GmbH, Vienna, Austria; Faculty of Medicine, Sigmund Freud University Vienna, Austria; Department of Neurology, St. John’s Hospital, Vienna, Austria; Karl Landsteiner University of Health Sciences, Krems, Austria; Division of Neurology, University Hospital St. Pölten, St. Pölten, Austria; Karl Landsteiner Institute of Clinical Neurology and Neuropsychology, University Hospital St. Pölten, St. Pölten, Austria; Karl Landsteiner University of Health Sciences, Krems, Austria; Institute of Laboratory Medicine (Central Laboratory), University Hospital St. Pölten, St. Pölten, Austria; Karl Landsteiner University of Health Sciences, Krems, Austria; Karl Landsteiner University of Health Sciences, Krems, Austria; Division of Neurology, University Hospital St. Pölten, St. Pölten, Austria; Karl Landsteiner Institute of Clinical Neurology and Neuropsychology, University Hospital St. Pölten, St. Pölten, Austria; Faculty of Medicine, Sigmund Freud University Vienna, Austria; Department of Neurology, St. John’s Hospital, Vienna, Austria

**Keywords:** posterior circulation stroke, thrombolysis, treatment, outcome, sex, onset-to-needle, time%

## Abstract

**Introduction:**

Posterior circulation (PC) stroke is underrepresented in most large-scale trials. While the importance of the onset-to-needle time (ONT) for intravenous thrombolysis (IVT) in anterior circulation stroke is well established, data on PC stroke are lacking. This study aimed to investigate how ONT affects functional outcome after IVT and to identify additional predictors of outcome in PC stroke.

**Patients and methods:**

IVT-treated PC stroke patients included in the nationwide Austrian Stroke Unit Registry between 2003 and 2024 were retrospectively analyzed. The primary outcome measure was the excellent (mRS 0–1) and non-excellent (mRS 2–6) functional outcome at 90 days. The secondary outcome measure was the occurrence of severe intracranial hemorrhage (sICH). Associations between ONT as continuous variable, clinical predictors, and functional outcomes were assessed using ordinal and binomial logistic regression models. A cut-off point for the transition from excellent to non-excellent outcome was determined by maximizing the odds ratio metric. The effect of ONT on sICH was analyzed dichotomously in time intervals of 0–150 min and 151–300 min.

**Results:**

Of 11,025 eligible patients with PC stroke, 1,359 (12.3%) were treated with IVT, resulting in more frequent excellent functional outcome in patients treated with IVT compared to best medical treatment (BMT) in the ordinal logistic regression (adjusted odds ratio (aOR) 1.31, 95% CI 1.16–1.47, *p* < 0.001). Correspondingly, binomial logistic regression showed fewer non-excellent functional outcomes in patients treated with IVT compared to BMT (aOR 0.73, 95% CI 0.63–0.85, *p* < 0.001). The odds of an excellent functional outcome were increased within the first 282 min, with a pronounced treatment benefit in the first 122 min. The transition cut-off point was found to be at 258 min. sICH occurred in 2.8% and was unrelated to ONT (aOR 1.28, 95% CI 0.55–2.91, *p* = 0.552). Overall, women had lower IVT rates (11.3% vs 13.0%, *p* = 0.007) and were more likely to experience a non-excellent outcome (aOR 1.31, 95% CI 1.19–1.45, *p* < 0.001), but had similar functional outcomes compared to men when treated with IVT (aOR 1.03, 95% CI 0.74–1.43, *p* = 0.883).

**Discussion and conclusion:**

A treatment benefit of IVT in PC stroke was observed within 4.5 h of stroke onset, with its maximum within the first 2 h. Women should receive special attention as they may be at a prognostic disadvantage due to lower IVT rates and less favorable overall outcomes.

## Graphical Abstract

**Graphical abstract:**
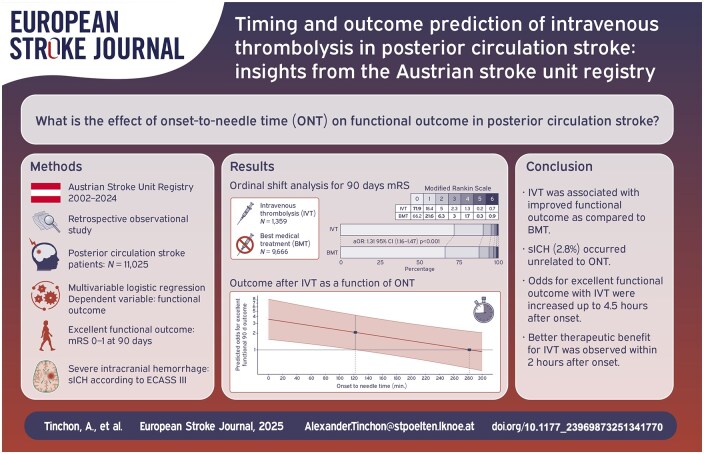

## Introduction

One in five strokes occurs in the posterior circulation (PC).^1^ This vascular territory is anatomically, radiologically, and clinically distinct from the anterior circulation (AC).^2^ Atypical clinical symptoms such as dizziness, gait instability, and oculomotor dysfunction can complicate stroke diagnosis.^2–4^ These symptoms often lead to an underestimation of stroke severity as they are inadequately assessed by the National Institute of Health Stroke Scale (NIHSS).^5,6^ Consequently, delays in both pre-hospital and in-hospital management have been observed, leading to prolonged treatment times for intravenous thrombolysis (IVT).^7–9^

Timely intervention is crucial in acute stroke care. In the AC, there is substantial evidence that clinical outcomes worsen as the time between symptom onset and treatment initiation increases.^10,11^ Comparative studies suggest that IVT in the PC yields clinical outcomes comparable to those in the AC, with a significantly lower incidence of hemorrhagic complications.^12,13^ However, the impact of treatment delays, as measured by onset-to-needle time (ONT) in the PC remains underexplored. This is clinically relevant because, due to a lack of data, existing imaging-based treatment decisions for AC stroke are difficult to apply to the PC, leaving time-based considerations as a key treatment decision marker.^14,15^ Furthermore, data on other predictors of clinical outcome following IVT in the PC are scarce due to limited sample sizes in the literature. This study aimed to investigate how ONT affects functional outcome and to identify additional outcome predictors for IVT in PC stroke.

## Methods

### Study design and data collection

The Austrian Stroke Unit Registry (ASUR), established in 2002 and funded by the Federal Ministry of Health, is a prospective database systematically collecting anonymized clinical data from stroke patients across all 39 stroke units in Austria. Data, including epidemiological details, clinical scores and assessments, are collected by experienced neurologists using a web-based platform immediately upon admission and discharge. To ensure high data quality and minimize entry errors, plausibility checks with standardized variables are implemented during and prior to the confirmation of patient records. Stroke syndromes are classified according to the Oxfordshire Community Stroke Project criteria.^16^ Further information on the methodology of the ASUR, including definitions of variables, scores and ratings, has been described previously.^17,18^ The inclusion of a patient with transient ischemic attack/stroke in the Stroke Registry is mandatory for financial reimbursement since 2018. Therefore, assuming that inclusion rates may have increased after 2018, we compared patient characteristics before and after 2018 and found no relevant demographic differences (Supplemental Table 1). A 3-months follow-up is facultative.

Patients with acute ischemic PC stroke aged >18 years entered the analysis. Those with basilar artery occlusion and/or treated with mechanical thrombectomy were excluded due to expected differences in clinical trajectories and functional outcomes. Follow-up modified Rankin Scale (mRS) was obtained during scheduled clinical visits or via telephone. To control for selection bias from missing follow-up, a comparative analysis was performed between patients with and without follow-up data at 90 days after stroke (Supplemental Table 2). The diagnosis of stroke was confirmed in the vast majority of cases by CT and/or MRI. In individual cases where cerebral imaging was negative or no MRI was performed, diagnosis relied on senior physicians’ assessments. IVT was performed with alteplase at the standard dose (0.9 mg/kg body weight, 10% as a bolus, 90% as an infusion over 1 h, max. 90 mg). Best medical treatment (BMT) included the administration of antiplatelet therapy with aspirin or dual antiplatelet therapy with aspirin and clopidogrel at the physician’s discretion.

### Outcomes

The primary outcome measure was the functional outcome at 90 days, defined as excellent (mRS 0–1) and non-excellent (mRS 2–6) functional outcome. The secondary outcome measure was the occurrence of severe intracranial hemorrhage (sICH) according to ECASS III criteria.^19^ Ordinal shift analysis was performed using ordinal logistic regression to assess the shift in mRS scores to determine the overall effect of IVT treatment.

### Statistical analysis

Results are presented as median and interquartile range (IQR) for continuous variables with non-normal distribution, as mean and standard deviation (SD) for continuous variables with normal distribution (age), while categorical variables are summarized by absolute frequencies (n) and relative frequencies (%). Descriptive comparisons of these characteristics were performed between IVT and BMT groups. Comparisons between categorical variables were performed using the χ2 test or Fisher exact test and presented as frequencies with percentages. The Kruskal-Wallis test was used for continuous variables. Statistical significance was set at *p* < 0.05.

Multivariable binomial logistic regression was performed to estimate the predicted odds and 95% confidence intervals (95% CI), providing the association between ONT as continuous variable and functional outcome. Independent variables included were: sex, NIHSS on admission, age, functional disability before stroke (defined as pre-mRS of 2–5), hypertension, diabetes, myocardial infarction, hypercholesterolemia, and atrial fibrillation. Within the process of data analysis, the VIF (Variance Inflation Factor) was used to test predictors for multicollinearity. As no variables with a VIF > 1.36 were identified, all predictors remained in the model. To assess the benefit of IVT on functional outcome, a multivariable logistic regression analysis was performed, with the ONT predictor replaced by the treatment variable (IVT vs BMT). For the analysis of bleeding risk associated with IVT, the ONT intervals were categorized dichotomously (0–150 min vs 151–300 min), given the low expected incidence of bleeding events. Statistical analyses were executed in R software (version 4.3.2).

An optimal cut-off time point at which the odds of a non-excellent outcome exceeded the odds of an excellent outcome was determined by maximizing the odds ratio metric. The Generalized Additive Models method was utilized to smooth (in this case, splines) the metric values across different cut-off points, ensuring a robust and stable estimation. The optimal cut-off point was identified as the value that maximized the smoothed odds ratio, providing the best separation between the two classes. Calculation of the optimization problem was conducted in R using the *cutpointr* function from the *cutpointr package* (version 1.1.2).

## Results

### Study population

**Figure 1.**
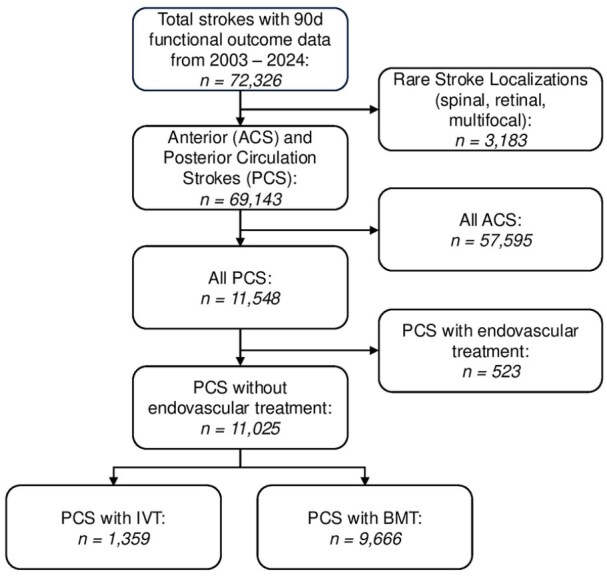
Flow chart of patient selection. ACS: anterior circulation strokes; PCS: posterior circulation Strokes; IVT: intravenous thrombolysis; BMT: best medical treatment.

Between 2003 and 2024, the ASUR recorded data on 200,405 acute stroke patients. In this study, 72,326 patients with available 90 days follow-up mRS (36.1%) were analyzed. After excluding 3,183 patients with rare stroke localizations (spinal, retinal, or multifocal), 69,143 patients remained, of whom 11,548 (16.7%) experienced PC strokes. Following the exclusion of 523 patients who underwent mechanical thrombectomy, 11,025 patients remained eligible for analysis, with 1,359 (12.3%) receiving IVT and 9,666 receiving BMT (Figure 1).

### Demographics

Baseline characteristics differed between IVT and BMT patients (Table 1). The IVT rates differed between sexes (13.0% in men vs 11.3% in women, *p* = 0.007). IVT patients presented with higher NIHSS scores (*4–10*: 48.1% IVT vs 22.5% BMT; *11–42*: 16.8% IVT vs 6.6% BMT) and higher sICH rates (2.8% IVT vs 0.8% BMT), whereas diabetes (25.0% BMT vs 20.0% IVT) and atrial fibrillation (21.5% BMT vs 16.9% IVT) were more common in BMT patients.

**Table 1.** Demographics of PC stroke patients.

Variable	BMT	IVT	*p*-Value
	*n* = 9,666	*n* = 1,359	
*Sex, n (%)*			**0.007**
Female	3,896 (40.3)	496 (36.5)	
Male	5,770 (59.7)	863 (63.5)	
*IVT rate by sex (%)*			**0.007**
Female	-	11.3	
Male	-	13.0	
*Age, mean (SD)*	69.1 (14.0)	69.1 (14.5)	
*n (%)*			0.869
18–54	1,625 (16.8)	237 (17.4)	
55–64	1,764 (18.2)	238 (17.5)	
65–74	2,567 (26.6)	366 (26.9)	
75+	3,710 (38,4)	518 (38.1)	
*NIHSS, median [IQR]*	2 [1, 4]	5 [3, 8]	
*n (%)*			**<0.001**
0–3	6,852 (70.9)	477 (35.1)	
4–10	2,176 (22.5)	654 (48.1)	
11–42	638 (6.6)	228 (16.8)	
*Vascular risk factors, n (%)*			
Hypercholesterolemia	5,813 (60.1)	831 (61.2)	0.205
Hypertension	7,425 (76.8)	1,027 (75.6)	0.475
Diabetes	2,420 (25.0)	272 (20.0)	**<0.001**
Atrial fibrillation	2,079 (21.5)	229 (16.9)	**<0.001**
Pre-stroke mRS 2–5	2,427 (25.6)	318 (23.4)	0.085
Myocardial infarction	8,59 (8.9)	135 (9.9)	0.146
*sICH, n (%)*	73 (0.8)	38 (2.8)	**<0.001**

NIHSS: National Institute of Health Stroke Scale; sICH: severe intracranial hemorrhage. Significant results are shown in bold.

Comparison of baseline characteristics between patients with BMT best medical treatment (BMT) and intravenous thrombolysis (IVT).

### Efficacy and safety of IVT

**Figure 2.**
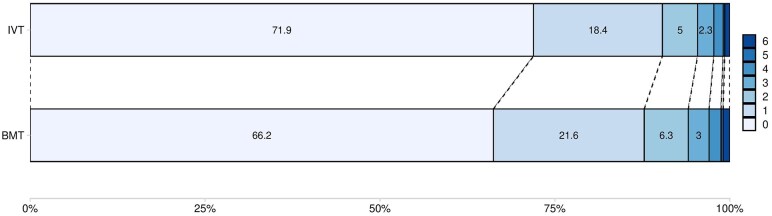
Ordinal Shift Analysis for 90 days mRS. Ordinal shift analysis of functional outcome after PC stroke based on ordinal regression analysis when comparing intravenous thrombolysis (IVT) to best medical treatment (BMT). The blue bars show the mRS scores from 0 to 6 and include the corresponding percentages for both treatment groups. For readability, mRS scores 4–6 are not shown in the graph. Intravenous thrombolysis (IVT): mRS 4: 1.3%, 5: 0.2%, 6: 0.7%. Best medical treatment (BMT): mRS 4: 1.7%, 5: 0.3%, 6: 0.9%.

Ordinal shift analysis showed better functional outcomes for patients treated with IVT compared to BMT (aOR 1.31, 95% CI 1.16–1.47, *p* < 0.001, Figure 2).

#### Functional outcome by ONT

**Figure 3.**
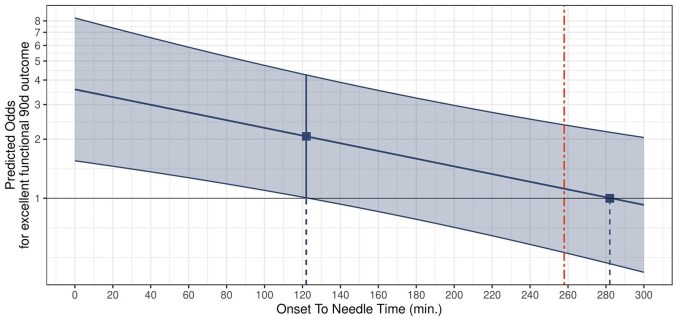
Outcome after IVT in PC stroke as a function of onset-to-needle time. Decrease in the odds of an excellent functional outcome (mRS 0–1) 90 days after PC stroke when onset-to-needle time is considered as a continuous variable. Red vertical line: calculated cut-off point for transition from excellent to non-excellent (mRS 2–6) functional outcome (258 min).

The odds of an excellent functional outcome after IVT decreased with longer ONTs. There was a time advantage defined by crossing confidence intervals up to 122 min after onset, which decreased continuously and was exhausted at 282 min. The calculated cut-off point beyond which the odds of a non-excellent functional outcome exceeded those of an excellent outcome was found to be at 258 min (Figure 3).

#### Functional outcome by demographic predictors

**Figure 4.**
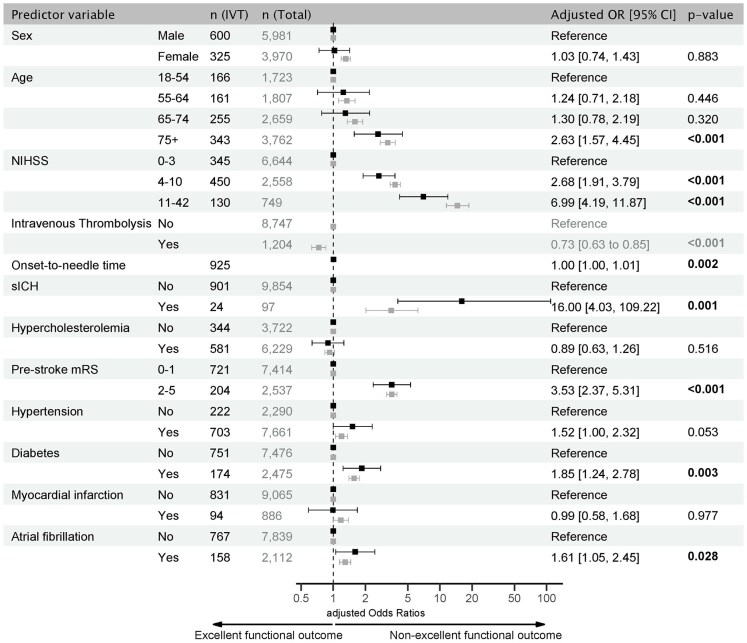
Forest Plot of predictors of functional outcome in PC stroke. Adjusted odds ratios (aOR) for non-excellent functional outcomes (mRS 2–6) 90 days after PC stroke based on multivariable logistic regression analysis. In black: IVT sample numbers (left column) and aOR with 95% CI. In gray: total sample numbers (right column) and aOR with 95% CI for comparison with the IVT sample (numerical values are provided in Supplemental Table 3). Significant results are shown in bold. CI: confidence interval; IVT: intravenous thrombolysis; sICH: severe intracranial hemorrhage; NIHSS: National Institute of Health Stroke Scale.

Besides ONT, older age (*75+ yrs*: aOR 2.63, 95% CI 1.57–4.45), increasing NIHSS (*4–10*: aOR 2.68, 95% CI 1.91–3.79; *11–42*: aOR 6.99, 95% CI 4.19–11.87), sICH (aOR 16.00, 95% CI 4.03–109.22), pre-stroke disability (aOR 3.53, 95% CI 2.37–5.31), diabetes (aOR 1.85, 95% CI 1.24–2.78), and atrial fibrillation (aOR 1.61, 95% CI 1.05–2.45) were associated with a non-excellent functional outcome after IVT (Figure 4). Consistent with the ordinal shift analysis, IVT was associated with fewer non-excellent functional outcomes compared to BMT (aOR 0.73, 95% CI 0.63–0.85, *p* < 0.001, Figure 4).

Predictors of non-excellent functional outcome in patients with IVT largely corresponded to those of the overall sample. Women had a worse functional outcome overall (aOR 1.31, 95% CI 1.19–1.45), but not when treated with IVT. In addition, the overall non-excellent functional outcome increased with each age group over 55 years (Supplemental Table 3).

#### Safety of IVT

**Figure 5.**
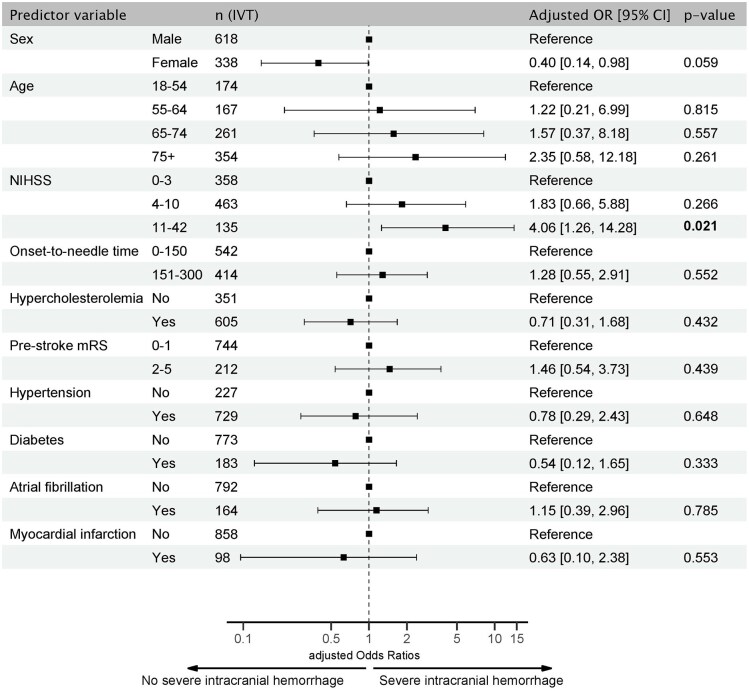
Forest Plot of predictors of severe intracranial hemorrhage after IVT in PC stroke. Adjusted odds ratios (aOR) for severe intracranial hemorrhage in IVT patients based on multivariable logistic regression analysis. Significant results are shown in bold. CI: confidence interval; IVT: intravenous thrombolysis; NIHSS: National Institute of Health Stroke Scale.

A total of 38 (2.8%) patients with sICH after IVT were observed. The incidence of sICH after IVT was unrelated to ONT. However, sICH was more frequent in patients with NIHSS scores ⩾ 11 (aOR, 4.06, 95% CI 1.26–14.28). The risk of bleeding tended to be lower in women (aOR 0.40, 95% CI 0.14–0.98, *p* = 0.059, Figure 5).

### Sex differences

In addition to having a lower IVT rate, women were older (*75+ yrs*: 48.0% female vs 32.0% male) and presented with higher NIHSS scores on admission compared to men (*11–42*: 9.9% female vs 6.6% male). Additionally, women were more likely to have a higher pre-stroke mRS (30.0% female vs 22.3% male) and atrial fibrillation (22.4% female vs 19.8% male), but had a lower prevalence of diabetes (22.1% female vs 25.8% male), hypercholesterolemia (57.0% female vs 61.9% male), and myocardial infarction (6.3% female vs 10.8% male) compared to men (Table 2).

**Table 2.** Demographics of PC stroke by sex.

Variable	Female	Male	*p*-Value
	*n* = 4,421	*n* = 6,677	
*IVT rate by sex (%)*	11.3	13.0	**0.007**
*Age, mean (SD)*	71.5 (14.6)	67.5 (13.5)	
*n (%)*			**<0.001**
18–54	641 (14.5)	1,228 (18.4)	
55–64	586 (13.3)	1,435 (21.5)	
65–74	1,070 (24.2)	1,878 (28.1)	
75+	2,124 (48.0)	2,136 (32.0)	
*NIHSS, median [IQR]*	2 [1, 5]	2 [1, 4]	
*n (%)*			**<0.001**
0–3	2,843 (64.3)	4,523 (67.7)	
4–10	1,139 (25.8)	1,711 (25.6)	
11–42	439 (9.9)	443 (6.6)	
*ONT in minutes, median [IQR]*	147 [105, 200]	140 [105, 193]	
*n (%)*			0.383
0–60	17 (4.6)	28 (4.2)	
61–90	48 (13.0)	96 (14.3)	
91–120	72 (19.6)	130 (19.4)	
121–150	65 (17.7)	150 (22.4)	
151–180	52 (14.1)	96 (14.3)	
181–210	51 (13.9)	64 (9.6)	
211–240	29 (7.9)	58 (8.7)	
241–270	26 (7.1)	35 (5.2)	
271–300	8 (2.2)	13 (1.9)	
*Vascular risk factors, n (%)*			
Hypercholesterolemia	2,520 (57.0)	4,131 (61.9)	**<0.001**
Hypertension	3,396 (76.8)	5,063 (75.8)	0.327
Diabetes	976 (22.1)	1,720 (25.8)	**<0.001**
Atrial fibrillation	992 (22.4)	1,321 (19.8)	**<0.001**
Pre-stroke mRS 2–5	1,325 (30.0)	1,490 (22.3)	**<0.001**
Myocardial infarction	279 (6.3)	718 (10.8)	**<0.001**
*sICH, n (%)*	39 (0.9)	72 (1.1)	0.308

IVT: intravenous thrombolysis; NIHSS: National Institute of Health Stroke Scale; ONT: onset-to-needle time; sICH: severe intracranial hemorrhage. Significant results are shown in bold.

Comparison of baseline characteristics between women and men.

## Discussion

Patients who received IVT had better functional outcomes than those who received BMT. Treatment initiated within 4.5 h after symptom onset was associated with increased odds of achieving an excellent functional outcome. Bleeding was rare and unrelated to ONTs. Women had a lower rate of IVT and a worse overall functional outcome than men, but benefited equally from IVT.

In the absence of dedicated randomized controlled trials and due to the limited representation of PC stroke in large treatment studies, current clinical practice in the management of PC strokes is extrapolated from data on AC strokes.^1,12^ In recent years, retrospective analyses have suggested comparable efficacy and lower bleeding rates for IVT in the PC compared with the AC.^12,13,20,21^ However, direct comparisons of IVT versus BMT in PC stroke are scarce.

In the AC, there is well-established evidence that the earlier IVT is administered, the better the clinical outcome. This is reflected in the golden hour concept, where outcomes are significantly improved if IVT is administered within the first 60 min after stroke onset.^10,22,23^ However, even beyond this window, time remains a critical prognostic factor.^11,24^

Two smaller retrospective analyses from China have addressed this issue in PC stroke. One study reported on 95 PC stroke patients in whom shorter ONTs did not affect functional outcome at 3 months, but improved recanalization rates within 24 h.^25^ Notably, this study included cases with basilar artery thrombosis, which accounted for almost half of the sample. Given the generally poor prognosis for these patients when treated without mechanical thrombectomy, it is likely that outcomes in these cases were not predominantly aligned with ONTs. Furthermore, basilar artery thrombosis is often associated with fluctuating levels of consciousness, complicating the precise determination of ONT. In addition, the study’s comparison across four time windows should be treated with caution due to the limited sample size.

Another study looked at 140 PC stroke patients within a 3-h window versus a 3–4.5-h window and found that short-term outcome, as measured by the NIHSS 24 h after IVT, was better in the earlier treated group.^26^ However, the NIHSS can be challenging as outcome parameter because it reflects clinical disability less accurately than the mRS, especially in the PC.^5,6^ Nevertheless, these findings suggest that time may indeed influence clinical outcomes after IVT in the PC.

Our study used large-scale registry data to delineate a treatment window for IVT based on ONT in PC stroke. As the focus of the analysis was on the effect of IVT on clinical outcome, we excluded mechanical thrombectomy and basilar artery occlusion, assuming different clinical trajectories. The most pronounced therapeutic benefit was seen when IVT was administered within the first 2 h. Although this should be interpreted as an exploratory estimate, it suggests that the decision to administer IVT should be made in a timely manner, despite the diagnostic challenges in the PC.

Analysis of ONT over time, along with the explored optimal cut-off point, indicates that 4.5 h marks a threshold beyond which the likelihood of a non-excellent outcome increases. This suggests that the optimal therapeutic window for IVT in the PC may parallel that of the AC. However, therapeutic urgency may be even greater in PC strokes due to typically longer ONTs, compared to AC strokes.^7–9,20^ For instance, with a median ONT reported at 175 min, the critical first 2 h of potential benefit would be largely elapsed.^7,20^ Fortunately, the risk of sICH remained low and appeared unaffected by ONT, suggesting that IVT should not be withheld, even in more advanced treatment times.

Beyond ONT, established demographic factors such as increasing age, initially high NIHSS scores, pre-existing disability, and well-known vascular risk factors such as diabetes and atrial fibrillation were associated with non-excellent functional outcomes after IVT in PC stroke.^27^ These predictors were largely consistent with those found in the overall sample, with the exception of sex.

Female sex was associated with worse functional outcomes overall. However, this disparity did not extend to women treated with IVT. The observation that female patients experienced less favorable clinical outcomes compared to male patients carries clinical implications, especially given the growing focus on sex-based differences in stroke research.^28^ In addition to older age and higher NIHSS scores on admission, the increased risk of non-excellent outcomes in female patients observed in this study may be due to lower IVT treatment rates.^29,30^ One possible explanation for their limited use of IVT may include older age and greater pre-stroke disability in women. However, recent findings indicate that women are more likely than men to present with non-specific stroke symptoms. Specifically, common symptoms associated with PC strokes, such as dizziness, gait disturbance and imbalance, are less frequently reported by women, which may increase the likelihood of missed stroke diagnoses and contribute to lower IVT rates among female patients.^29^ A large Korean registry study has recently confirmed these findings, showing reduced IVT administration in female stroke patients, consistent with evidence from large meta-analyses.^30–32^

Literature suggests that IVT outcomes may also be less favorable in women compared with men. However, regional factors may influence these sex-based differences, as most of these disparities have been documented in large European registry studies, with contrasting results in recent studies from China.^33–35^ Our data, which focus specifically on patients with PC stroke, do not suggest differences in outcomes after IVT between sexes. While caution is warranted in interpreting registry-based findings, the observed combination of lower IVT rates but comparable treatment benefits in women highlights the importance of incorporating sex-specific considerations into clinical practice and in the design of future prospective trials.

Several limitations have to be acknowledged. The retrospective and observational registry design introduces selection bias. However, as the systematic registration of stroke patients in the ASUR is mandatory in Austria, it can be assumed that the vast majority of strokes were included in this analysis. Moreover, the analysis was limited to an ONT of up to 300 min due to low case frequency thereafter, precluding investigation of longer treatment windows. Finally, there were missing follow-up data in our sample. To address this, we performed a group comparison between patients with and without follow-up, which revealed comparable baseline characteristics. We therefore considered our sample to be representative.

In conclusion, our findings suggest a treatment time window of approximately 4.5 h with most pronounced outcome benefit when IVT is initiated within 2 h after PC stroke symptom onset. IVT-induced sICH was rare and unrelated to ONT. In addition, as women may be at a disadvantage in terms of functional outcome, but appear to respond to IVT similarly to men, future studies should consider sex differences in optimizing treatment strategies for all patients.

## Supplementary Material

sj-docx-1-eso_23969873251341770

## Data Availability

Data that support the findings of this study are available from the corresponding author after national academic board review upon reasonable request.
