# Supplementary material for: Timing and outcome prediction of intravenous thrombolysis in posterior circulation stroke: Insights from the Austrian Stroke Unit Registry
Source: Eur Stroke J. 2026 Jan 1;11(1):23969873251341770. doi: 10.1093/esj/23969873251341770 (PMC12866274; doi:10.1093/esj/23969873251341770)
Supplement: sj-docx-1-eso_23969873251341770 [file sj-docx-1-eso_23969873251341770.docx]

Timing and Outcome Prediction of Intravenous Thrombolysis in Posterior Circulation Stroke: Insights from the Austrian Stroke Unit Registry

**Supplemental Material**

***Supplemental Table 1:*** Demographics of posterior circulation stroke patients for the time periods 2002-2018 and 2019-2024

***Supplemental Table 2:*** 90 days follow-up data of posterior circulation strokes

***Supplemental Table 3:*** Predictors of non-excellent 90 days functional outcome in PC stroke patients (total sample)

***Supplemental Table 1:*** Demographics of posterior circulation stroke patients for the time periods 2002-2018 and 2019-2024

|  | 2002 - 2018 | 2019 - 2024 |
| --- | --- | --- |
|  | n = 20,131 | n = 9,820 |
|  |  |  |
| *Sex (%)* |  |  |
| Female | 8,179 (40.6) | 3,900 (39.7) |
| Male | 11,952 (59.4) | 5,920 (60.3) |
|  |  |  |
| *Age, mean (SD)* | 68.4 (14.2) | 70.6 (13.7) |
| *n (%)* |  |  |
| 18 – 54 | 3,581 (17.8) | 1,346 (13.7) |
| 55 – 64 | 3,661 (18.2) | 1,807 (18.4) |
| 65 – 74 | 5,524 (27.4) | 2,456 (25.0) |
| 75+ | 7,365 (36.6) | 4,211 (42.9) |
|  |  |  |
| *NIHSS, median [IQR]* | 2 [1, 5] | 2 [1, 4] |
| *n (%)* |  |  |
| 0 – 3 | 12,678 (63.2) | 6,957 (71.0) |
| 4 – 10 | 5,664 (28.2) | 2,348 (24.0) |
| 11 – 42 | 1,720 (8.6) | 493 (5.0) |
|  |  |  |
| *IVT, n (%)* | 1,913 (10.2) | 1,510 (16.1) |
|  |  |  |
| *mRS at 90 days, n (%)* |  |  |
| 0-1 | 4,420 (58.9) | 2,179 (60.6) |
| 2-6 | 3,080 (41.1) | 1,419 (39.4) |
|  |  |  |
| ONT in minutes, *median [IQR]* | 145 [105, 195] | 150 [108, 210] |
| n (%) |  |  |
| 0 – 60 | 76 (5.0) | 36 (3.4) |
| 61 – 90 | 181 (11.9) | 126 (11.9) |
| 91 – 120 | 309 (20.2) | 211 (20.0) |
| 121 – 150 | 335 (22.0) | 190 (18.0) |
| 151 – 180 | 235 (15.4) | 165 (15.6) |
| 181 – 210 | 154 (10.1) | 127 (12.0) |
| 211 – 240 | 129 (8.5) | 107 (10.1) |
| 241 – 270 | 78 (5.1) | 72 (6.8) |
| 271 – 300 | 29 (1.9) | 23 (2.2) |
|  |  |  |
| *Vascular risk factors, n (%)* |  |  |
| Hypercholesterolemia | 10,557 (58.6) | 6,349 (71.5) |
| Hypertension | 14,538 (78.2) | 7,229 (78.6) |
| Diabetes | 4,632 (25.1) | 2,389 (26.6) |
| Atrial fibrillation | 3,955 (21.9) | 2,064 (22.1) |
| Pre-stroke mRS 2 – 5 | 5,407 (27.0) | 2,926 (29.9) |
| Myocardial infarction | 1,522 (8.3) | 827 (9.3) |
|  |  |  |
| *Affected brain area, n (%)* |  |  |
| Cerebellum | 5,279 (26.2) | 3,110 (31.7) |
| Brainstem | 8,937 (44.4) | 3,673 (37.5) |
| Occipital lobes | 5,915 (29.4) | 3,014 (30.8) |
|  |  |  |
| *sICH, n (%)* | 152 (0.8) | 75 (0.8) |

*Demographic comparison of patients in the years 2002-2018 and 2019-2024 (financial reimbursement period).*

IVT: Intravenous thrombolysis; NIHSS: National Institute of Health Stroke Scale; ONT: Onset-to-needle time; mRS: modified Rankin Scale; sICH: Severe intracranial hemorrhage; IQR: Interquartile range; SD: standard deviation.

Missing cases in the total number per category are due to incomplete data records for individual cases in the registry.

***Supplemental Table 2:*** 90 days follow-up data of posterior circulation strokes

|  | Complete | Missing |
| --- | --- | --- |
|  | n = 11,098 | n = 18,853 |
|  |  |  |
| *Sex (%)* |  |  |
| Female | 4,421 (39.8) | 7,658 (40.6) |
| Male | 6,677 (60.2) | 11,195 (59,4) |
|  |  |  |
| *Age, mean (SD)* | 69.1 (14.1) | 69.5 (13.9) |
| *n (%)* |  |  |
| 18 – 54 | 1,869 (16.8) | 3,058 (16.2) |
| 55 – 64 | 2,021 (18.2) | 3,447 (18.3) |
| 65 – 74 | 2,948 (26.6) | 5,032 (26.7) |
| 75+ | 4,260 (38.4) | 7,316 (38.8) |
|  |  |  |
| *NIHSS, median [IQR]* | 2 [1, 5] | 2 [1, 5] |
| *n (%)* |  |  |
| 0 – 3 | 7,356 (66.3) | 12,279 (65.4) |
| 4 – 10 | 2,850 (25.7) | 5,162 (27.5) |
| 11 – 42 | 882 (8.0) | 1,331 (7.1) |
|  |  |  |
| *IVT, n (%)* | 1,359 (12.3) | 2,064 (12.1) |
|  |  |  |
| *ONT in minutes, median [IQR]* | 142 [105, 195] | 150 [108, 201] |
| *n (%)* |  |  |
| 0 – 60 | 45 (4.3) | 67 (4.3) |
| 61 – 90 | 144 (13.9) | 163 (10.6) |
| 91 – 120 | 202 (19.5) | 318 (20.6) |
| 121 – 150 | 215 (20.7) | 310 (20.1) |
| 151 – 180 | 148 (14.3) | 252 (16.3) |
| 181 – 210 | 115 (11.1) | 166 (10.7) |
| 211 – 240 | 87 (8.4) | 149 (9.6) |
| 241 – 270 | 61 (5.9) | 89 (5.8) |
| 271 – 300 | 21 (2.0) | 31 (2.0) |
|  |  |  |
| *Vascular risk factors, n (%)* |  |  |
| Hypercholesterolemia | 6,651 (63.2) | 10,255 (62.6) |
| Hypertension | 8,459 (77.8) | 13,308 (78.7) |
| Diabetes | 2,696 (25.3) | 4,325 (25.8) |
| Atrial fibrillation | 2,313 (21.5) | 3,706 (22.3) |
| Pre-stroke mRS 2 – 5 | 2,815 (25.4) | 5,518 (29.5) |
| Myocardial infarction | 997 (9.4) | 1,352 (8.1) |
|  |  |  |
| *Affected brain area, n (%)* |  |  |
| Cerebellum | 3,135 (28.3) | 5,254 (27.9) |
| Brainstem | 4,815 (43.4) | 7,795 (41.4) |
| Occipital lobes | 3,137 (28.3) | 5,792 (30.7) |
|  |  |  |
| *sICH, n (%)* | 111 (1.0) | 116 (0.6) |

*Comparison of posterior circulation stroke patients with and without 90 days follow-up data.*

IVT: Intravenous thrombolysis; NIHSS: National Institute of Health Stroke Scale; ONT: Onset-to-needle time; sICH: Severe intracranial hemorrhage; IQR: Interquartile range; SD: standard deviation.

Missing cases in the total number per category are due to incomplete data records for individual cases in the registry.

***Supplemental Table 3:*** Predictors of non-excellent 90 days functional outcome in PC stroke patients (total sample)

|  | n | Adjusted OR | 95% CI |
| --- | --- | --- | --- |
| *Sex* |  |  |  |
| Male | 5,981 | Reference |  |
| Female | 3,970 | 1.31 | 1.19 – 1.45 |
|  |  |  |  |
| *Age* |  |  |  |
| 18 – 54 | 1,723 | Reference |  |
| 55 – 64 | 1,807 | 1.34 | 1.12 – 1.60 |
| 65 – 74 | 2,659 | 1.60 | 1.35 – 1.89 |
| 75+ | 3,762 | 3.24 | 2.76 – 3.82 |
|  |  |  |  |
| *NIHSS* |  |  |  |
| 0 – 3 | 6,644 | Reference |  |
| 4 – 10 | 2,558 | 3.80 | 3.41 – 4.25 |
| 11 – 42 | 749 | 14.59 | 11.49 – 18.72 |
|  |  |  |  |
| *IVT* |  |  |  |
| No | 8,747 | Reference |  |
| Yes | 1,204 | 0.73 | 0.63 – 0.85 |
|  |  |  |  |
| *sICH* |  |  |  |
| No | 9,854 | Reference |  |
| Yes | 97 | 3.50 | 2.02 – 6.22 |
|  |  |  |  |
| *Hypercholesterolemia* |  |  |  |
| No | 3,722 | Reference |  |
| Yes | 6,229 | 0.93 | 0.83 – 1.03 |
|  |  |  |  |
| *Hypertension* |  |  |  |
| No | 2,290 | Reference |  |
| Yes | 7,661 | 1.20 | 1.05 – 1.36 |
|  |  |  |  |
| *Diabetes* |  |  |  |
| No | 7,476 | Reference |  |
| Yes | 2,475 | 1.57 | 1.40 – 1.76 |
|  |  |  |  |
| *Atrial fibrillation* |  |  |  |
| No | 7,839 | Reference |  |
| Yes | 2,112 | 1.29 | 1.15 – 1.46 |
|  |  |  |  |
| *Pre-stroke mRS 2 - 5* |  |  |  |
| No | 7,417 | Reference |  |
| Yes | 2,537 | 3.54 | 3.16 – 3.96 |
|  |  |  |  |
| *Myocardial infarction* |  |  |  |
| No | 9,065 | Reference |  |
| Yes | 886 | 1.18 | 1.00 – 1.39 |

*Adjusted odds ratios for non-excellent functional outcome (mRS 2-6) 90 days after stroke.*

CI: Confidence interval; NIHSS: National Institute of Health Stroke Scale; IVT: Intravenous thrombolysis; sICH: Severe intracranial hemorrhage; mRS: Modified Rankin Scale.
